# Understanding coping strategies of mothers living with HIV who care for children living with HIV: a qualitative study in Indonesia

**DOI:** 10.1186/s12905-023-02299-y

**Published:** 2023-04-11

**Authors:** Nelsensius Klau Fauk, Hailay Abrha Gesesew, Lillian Mwanri, Karen Hawke, Maria Silvia Merry, Gregorius Abanit Asa, Paul Russell Ward

**Affiliations:** 1 Research Centre for Public Health, Equity and Human Flourishing, Torrens University Australia, Adelaide, South Australia Australia; 2 Institute of Resource Governance and Social Change, Kupang, Indonesia; 3 College of Health Sciences, Mekelle University, Mekelle, Tigray, Ethiopia; 4 Women and Kids theme, Aboriginal Health, South Australian Health and Medical Research Institute, Adelaide, Australia; 5 Medicine Faculty, Duta Wacana Christian University, Yogyakarta, Indonesia

**Keywords:** Coping strategies, Mothers living with HIV, Children living with HIV, Indonesia

## Abstract

HIV infection and its sequelae continue to be a significant challenge among women and their families in developing countries despite the progress that has been made in the prevention and treatment of HIV. This paper describes the strategies employed by mothers with HIV to cope with the various challenges experienced following their own and their children’s HIV diagnosis. This paper uses previously unpublished data collected for a study that sought to explore the mental health challenges and coping strategies of mothers living with HIV (MLHIV) (n = 23) who have children living with HIV (CLHIV). Data collection was conducted using in-depth interviews, and participants were recruited using the snowball sampling technique. The concept of meaning-making was used to guide the conceptualisation, analysis, and discussion of the findings. Our analysis showed that meaning-making such as the awareness of how important mothers were to their children/CLHIV and families and religious meaning were used by participants to cope with HIV-related and mental health challenges they faced. The meaning of mother-child relationship which was supported and maintained through the provision of time, attention and fulfillment of CLHIV’s needs were also coping strategies employed by these women. Additional coping strategies used were to link their CLHIV to groups and activities of CLHIV. The connections made through these links enabled their children to know other CLHIV, build relationships, and share experiences. These findings are useful evidence that can inform policies and practices and indicate the need for the development of intervention programs that address the needs of MLHIV and their families to cope with HIV-related challenges of their children. Future large-scale studies involving MLHIV who have CLHIV are recommended to have a comprehensive understanding of strategies they employ to cope with HIV-related challenging circumstances and mental health issues that they continue to face.

## Introduction

HIV infection has been reported to disproportionately affect women who represent over 53% of the total 37.7 million people living with HIV (PLHIV) globally [1]. In sub-Saharan Africa and Asia; and the Pacific regions, women represent 63% and 30%, respectively, of the total number of PLHIV [1–3]. In Indonesia, women represent 38% of the total of more than 400,000 PLHIV in the country [4, 5], which is higher than that of Asia and the Pacific region. One of the impacts of HIV that disproportionately affects women living with HIV (WLHIV) is mental health challenges, such as stress, anxiety, depression, fear, and sadness [6–9]. These psychological challenges are often triggered by various stressors including shame, a lack of social support, the advanced stages of their HIV infection, poor economic conditions, fear of death, and concerns about their children’s future [10–14].

Other HIV-related challenges experienced by WLHIV include stigma and discrimination within families, communities, and healthcare settings [6, 14–16]. WLHIV also experience the loss of employment or potential employments due to their HIV-positive status [17, 18] and poor physical strength that prevents them from working which in turn affects their economic condition [18–20]. Increased family expenditure for HIV care [18, 19] and forced sale of properties such as land and houses to cover the expenses for healthcare and daily needs, are also common HIV-related challenges that WLHIV and their families face [19, 21]. These challenges add to their precarious economic situation, leading to a vicious cycle of disadvantage, including food insecurity and amplified poverty [19–21]. Furthermore, forced or voluntary mother-child separation due to the fear of HIV transmission or inability to care for children [19–21] and rejection by a partner or spouse due to unacceptance of women’s HIV status are HIV challenges faced by WLHIV [17, 18, 21]. All these challenges in turn can lead to the deterioration of the psychological or mental health of WLHIV and family members [22–25].

To cope with mental health challenges, WLHIV employ different strategies which can be categorised at individual, interpersonal and institutional levels. At the individual level, acceptance of HIV status or self-acceptance, self-reliance [26–28], change in perception about HIV as a non-chronic disease [29] and feeling responsible for children [30, 31] have been used by WLHIV to cope with mental health challenges facing them. Research has found some individua-level coping strategies by WLHIV, such as coping for a ‘normal’ and positive life situation [27, 29], having an awareness that other people care for them and do not pose a threat to them [30], and perceiving low stigma and discrimination [31]. Additionally, WLHIV utilise spiritual coping strategies through prayers and religious practices [29, 32–34]. At the interpersonal level, WLHIV cope with HIV-related mental health challenges by seeking emotional, information, spiritual, financial, and material support from parents, siblings, partners, friends and religious leaders [26, 27, 30, 35]. There is also evidence for the benefits of peer group support with other PLHIV, through meetings and other peer group activities [28, 31, 36, 37]. At the organisational level, WLHIV cope through support from healthcare professionals and healthcare services [26], the church [38], and through utilising appropriate support systems for HIV-related financial assistance [30].

Although a range of HIV coping strategies have been used by WLHIV in different settings, there is a paucity of evidence globally on coping strategies used by mothers living with HIV (MLHIV) who also have children living with HIV (CLHIV). Whilst the broad literature on WLHIV may well have some overlaps with MLHIV (i.e., some of the WLHIV may also be MLHIV), there may be some very specific barriers, coping strategies and required policy/practice interventions for MLHIV who also have CLHIV. Also, there is a lack of evidence on HIV-related coping strategies used by both WLHIV and MLHIV in general in the context of Indonesia [39–41]. This study aims to fill in these gaps by exploring strategies used by MLHIV who have CLHIV in Yogyakarta, Indonesia to cope with HIV-related mental health challenges. Understanding how these mothers cope with mental health following their own and their child’s HIV diagnosis is necessary to inform HIV policies and develop intervention programs that address their needs and the needs of their CLHIV.

## Methods

### Theoretical framework

The concept of meaning-making was used to conceptualise the study, analyse and discuss the findings [42]. The concept of meaning-making is understood as a psychological process that explains the extent to which people have made sense of or found meaning from experiencing challenging circumstances or stressful events [36, 42]. In this study, we used this concept to guide an understanding of how MLHIV who have CLHIV find the meaning in difficult situations related to their own and their child’s HIV diagnosis, which helps them to cope with the mental health challenges they experience. This concept suggests that people in challenging life situations usually try to identify causal attributions of the situations or events, appraise and find the meaning of the events [36, 42]. If the meaning is congruent with their goals or beliefs, such as belief about self-worth (e.g., in this case, the mothers’ belief about their importance and responsibility for their children and families) then people can handle the challenging situations or events. This will lead to their acceptance of the challenging life situations, enabling them to cope with them [36, 42]. However, if the meaning is incongruent against their goals or beliefs, people will reappraise the meaning of the challenging situations and their goals or beliefs, in which may lead to changes that can help them cope with the situation [42]. Therefore, the meaning people find out of any challenging circumstances or stressful events can be both a process and an outcome of coping [42].

### Recruitment of the participants, data collection, and ethical considerations

As part of a larger qualitative study exploring the mental health challenges, the associated factors and coping strategies used by MLHIV (n = 23) who have CLHIV in Yogyakarta, Indonesia [43], this paper describes strategies employed by these mothers to cope with mental health challenges experienced following their own and their child’s HIV diagnosis. Yogyakarta was selected as: (a) there is a paucity of evidence on the mental health challenges facing MLHIV who have CLHIV and the strategies used to cope with these challenges in the current study setting, and (b) the familiarity and feasibility so as to potentially undertaking the study successfully. Study participants were recruited using the snowball sampling technique. After an initial conversation with the head of a HIV clinic and a buddy of PLHIV (also known as *pendamping Orang Dengan HIV/AIDS* or *ODHA*) in the study setting, the study information sheets were distributed to potential participants through both the information board at the clinic and a WhatsApp group of PLHIV. Potential participants who contacted the researchers to confirm their participation were recruited and interviewed. At the end of the interviews, participants were also asked to disseminate the study information sheets to other MLHIV. Only MLHV, aged 18 years or above, and had a child or children living with HIV were recruited to participate in the study. A total of 23 MLHIV were finally recruited. Each of them had only one child living with HIV and nine had additional child or children who were HIV free.

Participants ages ranged between 24 and 43 years. The majority (65%) were employed, while eight (35%) were unemployed or housewives. Fifteen mothers were married or re-married and eight were widowed or never married. The mothers had been diagnosed with HIV for different periods of time, from 1 to 5 years (n = 7), 6–10 years (n = 12), and 11–15 years (n = 4). Their CLHIV were between 1 and 5 years old (n = 9), 6–10 years old (n = 11) and 11–15 years (n = 4). Six children were in kindergarten, ten in primary school, and two in junior high school, while five had not started school. All the mothers and their CLHIV were on HIV treatment at the time this study was conducted.

Data collection was carried out by two researchers (NKF resides outside of Yogyakarta; MSM lives in Yogyakarta) using in-depth interviews. NKF is a full-time researcher at Torrens University Australia and has a PhD qualification in Public Health. He has attended formal training in qualitative methods and conducts research on various public health issues, including HIV/AIDS, mental health, and disability with different marginalised groups. MSM is a physician and senior lecturer. She attended formal training in qualitative and quantitative methods and has more than ten years of work experience in the field of HIV in different settings in Indonesia. The interviews were conducted either in person or online using WhatsApp video calls, based on the preference of each participant. Because of COVID19 pandemic, we aimed to provide participants with options including online interviews through WhatsApp video calls which are popularly used in Indonesia to increase the possibility of participation and for the safety of both the researchers and participants. Only a field researcher and each participant were present in the interview room (or on the screen). An interview guide was used in the interviews and in regards to coping strategies, the interviews mainly focused on understanding participants’ perceptions and experiences of how they had been coping with mental health challenges following their own and their child’s HIV diagnosis. The examples of some main interview questions are: (i) What is your perception and feeling about how important you are to your children, especially their CLHIV, and families? (ii) Could explain more about your responsibility for your children and families and how do you manage to do it? (iii) What is your religious perception and experience following your own and your child’s HIV diagnosis? (iv) How would you explain your relationships with your CLHIV? (v) What have you done to support their CLHIV? (vi) Do you think all these help you in coping with mental health challenges facing you and how?

Interviews were conducted in Indonesian (also known as Bahasa Indonesia), the national language of Indonesia, and took approximately 40 to 60 min. The interviews were recorded using a digital tape recorder and notes were also undertaken during the interviews. Data analysis was ongoing, beginning after the first interview and continuing throughout data collection and post data collection. Data collection ceased after 23 interviews (data saturation) since ongoing analysis found that the information provided by participants was detailed enough to address the research questions and objectives and the same or similar information was emerging in the final few interviews. To maintain confidentiality and anonymity, we assigned a pseudonym to each interview transcript, and all participants were informed about this. Before the interviews, we also made sure that the participants understood their rights to participate or not to participate, and knew that they could withdraw their participation at any time without any consequences. An informed consent form was provided and signed by all the participants to indicate their agreement to voluntarily participate in this study. They returned the signed form to the researcher before commencing the interviews either in person or through WhatsApp.

>Ethics approval for this study was obtained from Health Research Ethics Committee, Duta Wacana Christian University, Yogyakarta, Indonesia (No. 1005/C.16/FK/2019). The methods used in this study were performed in accordance with the relevant guidelines and regulations.

### Data analysis

To enable comprehensive analysis of the data, all the recordings were transcribed verbatim and manually into coding sheets. Five steps of qualitative data analysis were followed during the entire process of data analysis [44, 45], including: (i) familiarisation with the data, where the transcript of each interview was read to familiarise with the information provided by each participant. This was an iterative process through which comments and labels to chunks of information or data extracts of each interview were provided; (ii) identification of a thematic framework, where key issues, concepts, and ideas from each transcript were identified and listed. They were then defined and refined throughout the data analysis process and used to form a thematic framework. Concepts from the conceptual framework on ‘meaning-making’ were critical here since data were analysed in terms of if/how/why they related to meaning-making; (iii) indexing the data where the entire data were then indexed by providing codes to data extracts in each transcript. These codes were then listed to identify similar or redundant codes and collate them, and codes that formed a theme were grouped under the same theme: family meaning, mother-child support and relationship meaning, social meaning for CLHIV, and religious meaning; (iv) charting the data by reorganising and rearranging the themes and their codes together in a summary chart to enable comparison of the data within each interview or across interviews; and (v) mapping and interpretation of the data as a whole [44, 45], and in particular, the relationships between our data and the concepts within the literature on meaning-making. Cross check and comparison of the data or transcripts were performed by NKF and MSM during the transcription process to maintain the quality and validity of the data. Data analysis was carried out in Indonesian to maintain the social and cultural meanings attached to verbal expression from participants and for publication purposes, the selected quotes were translated into English by NKF and verified by MSM. Data were further checked for clarity of transcription and accuracy of the translation by other authors. Team-based discussions of the findings were undertaken during the analysis and writing stages, and team decisions were made about the validity of the final themes and interpretation.

## Results

Utilising the meaning-making framework described above [42] provided a robust understanding of the mechanisms that fostered coping for these women. Four themes were identified from participants’ narratives and these have been expanded below.

### Family meaning: the importance of the mothers to their children and families

The awareness of how important mothers were to their families, including their responsibility to the CLHIV was prevalent among participants. For most participants, the importance of being mothers in families held significant meaning, and as one of the strategies employed to cope with challenging HIV-related circumstances. One MLHIV said, *“If I don’t fight this infection and stay strong then who will take care of my children, especially XX who is also sick [HIV-positive]”* (Erna). This sense of responsibility encouraged them to fight against or cope with fear, stress, anxiety, depression, and other negative feelings and experiences facing them following their own and their child’s HIV diagnosis:

*“As I said, after the [HIV] test I was stressed out, scared, and could not sleep. These were even worse once I found out that my daughter was also infected with HIV. I was anxious all the time. I was so much depressed. After a while, I started to realise that my family: my kids, especially the one who is also sick (HIV+), and my husband (also HIV+) need me. They need my presence and support. This kind of awareness helped me fight against the fear, stress, and depression I have been facing. I have been trying so hard to focus on my children’s health, life, and future. At least it helps me stay strong up to now. But don’t get me wrong, these feelings do not completely go away, sometimes I still feel stressed, worried, guilty, or overthink”* (Ika).

Understanding how important their role as a mother was to their family, also assisted the women to gain acceptance around their own HIV status, and the HIV status of their child. A 27-year-old widowed MLHIV said, “*I was so depressed, especially after my son was diagnosed with HIV, but after a while, I realised that my son needs me and I have to accept this condition to move on*” (Marni). Similarly, the stories of the participants showed that the awareness of their responsibility for their children and the acceptance of their HIV status and the HIV status of their child helped them put aside negative feelings or mental health challenges and focus on HIV treatment by regularly accessing antiretroviral therapy for themselves and their CLHIV, and adhering to the therapy:

“*After my daughter was diagnosed with HIV, I pulled myself together and thought that I have to be strong for her sake even though my heart was broken into pieces. Those days were the most painful days in my life. I cried a lot. But I always tried to think positively and told myself that this is the kind of life that I have to live for the rest of my life. So, day by day I started to make peace with the condition (having HIV infection) and accept it. These have helped me to prioritise my children, and undergo the treatment for me and my daughter. So, both of us have been on HIV treatment up to now”* (Ima).

This acceptance was in itself, a useful strategy to stop the feelings of self-blame, and blaming a partner or spouse. Participants described that following their own and their child’s HIV diagnosis they started blaming themselves due to negative behaviours they had engaged in or blamed their partner or spouse who transmitted the virus to them. Such attitudes were acknowledged to elicit anger, stress and rejection of their condition and the condition of their child, which in turn, prevented them from seeking social support, and HIV treatment. By recognising that CLHIV and other family members look to the matriarch of the family for support and guidance, and that focusing on the anger and blame was not actually going to change their situation, women reported being able to let much of these negative emotions go, and focus on accessing support for themselves and their family:

*“I was scared once the doctor told me that I have HIV but what I felt once my daughter tested positive was so painful. It hurts me even until now. In the beginning, I did blame myself for what I have done. I used injecting drugs and engaged in group sex, so I got HIV. My husband is also positive. But when we started accepting our condition, things started to change in a positive way, and now we accept everything and try to live a healthy life. No blaming anymore”* (Dewi).

“*I have accepted my condition and my son’s condition and thrown away all bad memories in the past. I used to blame my husband (who died from AIDS) a lot because he transmitted HIV to me. He knew that he was infected but hid it from me and transmitted it to me. Every time we talked, I blamed him and got so mad at him. I felt anger and stress inside me towards him. But once I started accepting our condition, I can cope with the stress and anger, calm down myself and can focus on helping myself and my son, accessing healthcare and social support for us”* (Silvia).

### Religious meaning: The importance of faith and prayer

Spirituality and faith were reported as important determinants of coping mechanisms for the myriad of challenges MLHIV faced. Praying was reported as a useful strategy, especially with accepting a HIV diagnosis. Participants described how praying to God helped them calm down, think positively and gave them hope for a better future. They believed that God is in control over everything and acknowledged that through prayers, they ask for mercy and help, and could openly share their feelings, experiences and burdens with God. The following narratives of two MLHIV reflect the meaning of prayer and their relationships with God, and how these helped them cope with mental health challenges they went through after they and their child tested positive for HIV:

*“When my daughter tested positive for HIV, my life was very dark. I was stressed, anxious, scared, and sad all the time. I was really broken due to this very sad situation. I began to slowly pray diligently. In my prayer, I kind of confide in God, I say everything that I feel and the bad experiences I’ve had. I surrender completely to God. ‘God, here I am, I am guilty and now my husband, daughter and I are sick, please help us’. Every time I pray to God I feel calm, that’s why I keep doing it until now. It gives peace to my heart”* (Ayu).

*“Prayer is one of the things that make me feel calm and think positively about the situation that my son and I, as well as my family, are going through. I believe everything is in God’s hands. So, I keep trying to get closer to God through my prayer and keep positive thoughts about my life, my children, and my family”* (Julia).

### The meaning of the mother-child relationship: strengthening familial bonds

Strengthening the relationship between mother and child after HIV diagnosis, was a significant coping tool reported by the participants. All participants acknowledged that transmitting HIV to their child or receiving a HIV diagnosis for their child increased the already existing burden of stress, depression, anxiety, fear and guilt they felt following their own HIV diagnosis These feelings were directed toward not only their CLHIV, but also their HIV-negative children, who they felt had also borne the brunt of these diagnoses. By providing more time and attention to their children and supporting their needs, the women reported feeling a reduction in the burden of guilt they felt. For example, a 25-year-old single mother who had two children (one is HIV+) said “*I want to give more of my time and attention to my kids, especially my son [living with HIV]. I transmitted HIV to him and he has to carry the burden as well, he doesn’t deserve this*” (Yeni). These time and attention were reflected in the reported mother-child collective activities such as playing or hanging out, or going to recreational places together:

*“I feel guilty towards my daughter because of infection. We both don’t deserve it. I got it from her father and transmitted it to her as well. I don’t care about myself because I am old enough to carry this but she is still little. So now, she has my full attention, my time is for her. I want her to feel that she has my support, that is what I have been trying to build for both of us. I try to make her happy. I accompany her wherever she wants to go, sometimes she wants to play with her friends, go around the shopping centre, or go to the beach. These make me feel good, at least these reduced the burden and guilty feeling I have towards her”* (Indah).

Fulfilling the needs and wants of their CLHIV was very important to the mothers, and helped them to feel better and reduce their feelings of guilt Several participants with incomes described that they tried as much as they could to fulfill the needs of their children, especially the ones living with HIV, or to indulge what their children wanted. These were clearly described in their stories as illustrated in the following narrative of a 37-year-old mother of two children, and who had a stable monthly income:

*“My husband (also HIV+) and I always try to provide for our children. If they want to eat this or that then I will definitely buy it. Also, if they want new clothes, new shoes, and new toys, I’ll definitely buy them. As a parent, I feel guilty because they have to bear the consequences of HIV infection, especially XX who also got infected with HIV. If they are happy then I am also happy and can forget all kinds of negative feelings”* (Astri).

Providing for their children and giving them all the support, love and material things, felt very meaningful for mothers, and strengthened their relationship with their children, especially the CLHIV. This was reflected in narratives like: *“The meaningful aspect I learn from this situation is that my son and I are now very close because we always go everywhere together, play together, eat together*” (Lina), *“My daughter (HIV+) and I are inseparable. I feel that we are very close since she was tested positive”* (Jeane). Moreover, some mothers described what they did for their children also made them feel each other’s care and love:

*“Ever since my daughter was diagnosed, the two of us have been kind of inseparable. I always try to be there for her. When I come home from work, I always make time to play and talk with her, ask her what she wants, or if she experienced anything at school or with his friends. I pay attention to every little thing about my daughter. I love her and feel her love for me too. Sometimes she asks whether I have already eaten or not, or have taken my medicine or not. I can feel her care and love and I feel happy about it. The situation we are facing brings us to such a very meaningful relationship and that is a very good thing for me and my daughter”* (Anti).

The meaning of social interaction and support for CLHIV: Bringing CLHIV together through groups and activities.

Linking CLHIV to other CLHIV through groups and activities was a productive strategy used by MLHIV to cope with feelings of worry and fear about the possibility of their child’s HIV status being exposed. Some described: *“I bring my child to group (of CLHIV) because I am not worried and afraid that my child’s HIV status will be found out when he plays with his (HIV+) peers. They are all the same (HIV+)”* (Fatima). These CLHIV groups, which are coordinated by buddies of PLHIV, encouraged the children to engage in activities and competitions. Positive interaction with other CLHIV was reported as a real sense of relief for the mothers, who were often worried about their child being exposed to bullying, stigma and discrimination by HIV-negative children and adults. Many children in the group were unaware of their own HIV status, though all adults including the group coordinators understood it was a group specifically for CLHIV. This was reflected in the following quote:

*“I put my son in a group of children living with HIV because they are coordinated by the companions of PLHIV, and there are various activities and competitions with prizes for them. I don’t feel anxious or afraid if my child plays with his peers who are also HIV-positive because I think there are definitely no possible negative impacts such as being bullied, avoided, or stigmatised by other kids. They are all HIV-positive although in general most of them do not know about their HIV status”* (Ratih).

Joining and participating in those groups and activities provided opportunities for the children to have social relationships with their peers; children who are going through a similar experience. Mothers perceived these interactions as socially meaningful for their CLHIV and often thought ahead to the future, where in time their CLHIV will become aware of their own HIV status and will experience a range of emotions and feelings about that. They felt that having peers that are going through the same experience would be beneficial to share stories, feelings, and experiences, and support each other. This seemed to help the mothers cope with the burden of overthinking and worrying about possible future difficulties and negative social impacts. Thus, participation in these groups and activities was a proactive strategy to create resilience and a robust social support system that will prepare and nurture CLHIV through their childhood and beyond:

*“One thing that makes me worried too much is my concern about what might happen to my daughter in the future. How she will cope with the fact that she has HIV once she grows up and is informed about it. I am glad that by joining the group of children living with HIV and participating in activities with other kids she has many friends who are also living with HIV. She has social life and friends who can understand her in the future once they grow up and know about their HIV status. I think this is very positive for her and her social life in the future. It is a kind of relief for me because I think they will group up together and support each other as they are going through the same experience of living with HIV”* (Retno).

In Indonesia, it is normal to have regular treatment regardless of whether the child is aware of their HIV status. After attending group sessions, mothers reported to have noticed a reduction in their children’s questioning and complaints about why they had to take medication, and what it was for. With this reduction, mothers reported a sense of relief when hearing their children talk to each other about their regular HIV medicine in group sessions, and could see that it was beneficial for their CLHIV to hear from their peers.

*“I was so happy knowing that my daughter and her friend (who is also HIV+) talked about their experience of taking medicine every day. She once told me about it: ‘Mom, my friend XX told me that she takes medicine every day before going to sleep, the same as me, ….’. Both of us talked a lot about it and it was a kind of relief for me because I think at least she knows that she is not the only one who takes medicine regularly. After that, she doesn’t complain that much anymore as she used to”* (Enda).

## Discussion

A HIV diagnosis has a range of negative impacts on women, including mental health challenges [6, 14, 16, 46, 47], and it is plausible to state that the complexities of navigating how to live with CLHIV would predispose women to extraordinary stress. This study aimed to understand strategies used to cope with mental health challenges experienced following a HIV diagnosis by MLHIV who care for their CLHIV in Yogyakarta, Indonesia. In the current study, a number of identified coping strategies employed by MLHIV were consistent with the findings of the past research [36, 42]. These included MLHIV’s strong efforts to make sense of their own HIV status and having their child diagnosed with HIV and to construct the meaning of themselves and their role to their family, especially their CLHIV. This meaning-making framework helped them to make peace within themselves and ultimately find positive coping strategies for mental health challenges. These findings are in line with the concept of meaning-making or people’s belief about their self-worth through self-evaluation of their goodness and importance [42]. The findings are also consistent with studies that report the awareness of WLHIV about how meaningful they are to their children, families, and significant others help them cope with mental health challenges experienced after a HIV diagnosis [30, 31, 36].

As has been reported in broader literature on WLHIV, prayers were used by MLHIV to mitigate the impact of mental health challenge of HIV through sharing their feelings, experiences and burdens with God. This was acknowledged as calming and helpful to positively cope with stress, depression, anxiety and guilt. Such religious meaning-making seemed to stem from mothers’ strong beliefs in God, and God was said to be the One in control over everything, thereby taking some of the burdens off the mothers’ shoulders, and allowing them to hope for a better future for their CLHIV [26, 29, 33, 36]. Previous studies have found that self-acceptance of HIV status and self-reliance are strategies used by WLHIV to cope with their difficult experiences of HIV-related mental health issues [26–28, 36]. Similar findings were also identified in our study. The acceptance by MLHIV of their own and their children’s HIV status helped them to move past the feelings of helplessness, guilt and stress, and in finding a healthier way of living with HIV for themselves and their children. Our study provides additional evidence, suggesting that this acceptance of HIV status helped mothers let go of negative attitudes and resentments toward themselves and their partners or spouses. With letting go of guilt or negative attitudes, they do shift their focus to health promoting attitudes and behaviours, including accessing and adhering to antiretroviral therapy for themselves and their CLHIV. It is therefore reasonable to allude that because of letting go of the feelings of negativity which can cause a person to have self-doubt, frustration, anger, and which may result in furthering stress and poor mental health, the ability of letting go may then help the MLHIV and their children to improve, not only the mental health, but theirs and their families’ overall health and wellbeing.

Our study adds new coping strategies used by MLHIV, that are as yet to be reported previously with WLHIV in general [32, 34, 37, 48]. MLHIV’s focus and perseverance on building mother-CLHIV support and strengthening their relationship through increased time and attention, and fulfilling needs and demands, were reported as useful strategies to reduce feelings of stress and guilt. The use of these strategies was initially reported to benefit the mothers by alleviating their feelings of stress and worry. However, it apparent that there were greater benefits for their children, including bringing happiness, closeness, and a strengthened bond between mother and child. These coping strategies also reflect the meaning-making concept as the mothers were able to identify causal attributions of the challenges they and their children faced, using this newly found knowledge to portray positive and supportive attitudes and behaviours for their CLHIV [36, 42]. It is therefore reasonable to hypothesis that with the newly found positive attitudes, MLHIV’s state of mind would be to envision and expect good prospects regardless of the negative situations that would surround them, and in turn, improve their happiness, health, and expectation of much more favourable outcomes.

Linking CLHIV with other CLHIV through groups and activities was another novel strategy found to have been used by mothers in the current study, which has not been reported in previous studies with WLHIV [32, 33, 37]. The connections between their own CLHIV and other CLHIV seemed to have normalised the HIV positive status and reduced the mothers’ fears of the disclosure of their child’s HIV status. The disclosure of the HIV positive status, which would have been perceived to cause negative consequences for their CLHIV was now an easier thing to do which further alleviated stress, leading to improved mental health outcomes related to a fear of the HIV status disclosure. Therefore, participation of their CLHIV in groups and activities with their peers who are also living with HIV was considered unharmful, and positive. In addition, participation of their CLHIV in such groups and activities was also considered socially meaningful for the children and as an opportunity for them to get acquainted or start building social relationships with other children who have the same experience of living with HIV. This also helped the children to feel positive about themselves, accept HIV treatment and reduce treatment-related questions and complaints which were the sources of stress and guilty feeling facing MLHIV.

### Strengths and limitations of the study

The strength of this study is that it involved MLHIV who have CLHIV, which is a specific group that to our knowledge had not been a focus of previous studies exploring HIV-related mental health challenges and coping strategies. Thus, our findings have significant implications for both health and non-health sectors in Yogyakarta and other similar settings in Indonesia and beyond. The information may be useful to address HIV issues for mothers and women living with HIV in general, through policy and practice change that specifically targets women such as those who participated in the current study. The snowball sampling technique used to recruit participants could be a limitation as this might have led to recruiting participants from the same networks of mothers who had been on antiretroviral therapy and who were seeking support. As a consequence, we might have missed different perspectives or experiences about HIV-related coping strategies from MLHIV who are disengaged in HIV treatment and care. Interpretations of the current findings need to consider these limitations as the findings mainly reflect the perspectives and specific experiences of participants in this study.

## Conclusions

This study presents several strategies used by MLHIV who have CLHIV to cope with mental health challenges after their own and their child’s HIV diagnosis. Meaning-making such as the awareness of how important a mother’s role is in the life of children and families, the religious meaning they believed in, the bond between mother-CLHIV, and the social meaning and impact on peer-relationships for CLHIV were significant strategies used to cope with difficult situations, feelings, thoughts, and mental health challenges. Future large-scale studies are recommended to gain a comprehensive understanding of coping strategies MLHIV and/or mothers of CLHIV employ to positively move forward post HIV diagnosis. Findings from larger studies could steer future development of evidence-based policies and practices to support women and children living with HIV globally.

## Data Availability

The data cannot be shared publicly because of sensitive information regarding the interviewees and the restriction set by the human research ethics committees. Data presented in this study are available on request from the corresponding author for researchers who meet the criteria for access to confidential data.

## Abbreviations

CLHIV: Children Living with HIV
HIV: Human Immunodeficiency Virus
MLHIV: Mothers Living with HIV
PLHIV: People Living with HIV
WLHIV: Women Living with HIV
